# Optimizing neonatal management in the prescence of intrapartum maternal fever via the integration of an early-onset sepsis risk calculator and dynamic inflammatory markers

**DOI:** 10.3389/fped.2026.1670738

**Published:** 2026-02-11

**Authors:** Bin Wu, Jiemin Liu, Xuemei Fu

**Affiliations:** Departments of Neonatology, International Peace Maternity & Child Health Hospital, Shanghai Jiao Tong University School of Medicine, Shanghai, China

**Keywords:** biomarker, early-onset sepsis, early-onset sepsis calculator, maternal intrapartum fever, neonate

## Abstract

**Background:**

Intrapartum maternal fever is associated with an increased risk of early-onset sepsis (EOS) in infants. However, EOS has a high mortality rate, and its clinical symptoms are variable and often atypical, posing significant challenges for early identification and treatment. This study aims to evaluate the performance of an EOS risk calculator in combination with inflammatory markers for predicting EOS in infants born to mothers with intrapartum fever while maintaining neonatal safety in China.

**Methods:**

This was a retrospective cohort study involving 265 term neonates (gestational age: 37–41⁶/₇ weeks) born between the 1st of January 2024 and the 30th of June at the International Peace Maternity and Child Health Hospital. Eligible neonates were admitted to the neonatal intensive care unit (NICU) due to a intrapartum maternal fever (temperature >37.5 °C). We collared and reviewed laboratory data and medical charts of all included neonates. EOS risk scores for all neonates were calculated using the EOS risk calculator, and the results were analyzed and compared with blood biomarkers.

**Results:**

Of the 265 neonates, 61 (23.0%) were diagnosed with EOS. Among the 204 (77.0%) low-risk newborns predicted by the EOS risk calculator, none developed culture-confirmed sepsis. Infected neonates exhibited significantly higher levels (*P* < 0.01) of inflammatory markers, including C-reactive protein (CRP) and Interleukin 6 (IL-6), compared to uninfected counterparts. Receiver Operating Characteristic (ROC) Curve analysis revealed that when the EOS risk calculator was combined with inflammatory markers, CRP had the highest discriminative capacity [Area Under Curve (AUC): 0.84], while IL-6 exhibited moderate accuracy (AUC: 0.72). The combination of CRP and IL-6 further improved diagnostic performance (AUC: 0.85).

**Conclusions:**

Combined with inflammatory markers, the EOS risk calculator demonstrated good predictive accuracy and a high safety profile for the clinical management of neonates with presumed sepsis, which helps reduce antibiotic use and promotes rational use of medical resources.

## 1 Introduction

Intrapartum maternal fever complicates 5%–10% of all deliveries globally (1) posing significant interdisciplinary challenges for obstetrics, anesthesiology and neonatology teams. This condition is associated with a 2- to 4-fold increased risk of admission to the neonatal intensive care unit (NICU) (2) along with a range of maternal complications, including increased rates of cesarean delivery and operative vaginal delivery, and postpartum hemorrhage. Chorioamnionitis (CA), accounting for 30%–50% of intrapartum fevers (3), independently elevates the risk of early-onset sepsis (EOS), which is a systemic inflammatory response syndrome in newborns caused by fungi or bacteria and is a major cause of morbidity and mortality among infants by 3- to 5-fold in neonates (4, 5). With 1%–2% of exposed neonates developing culture-confirmed EOS (6), systematic risk stratification is critical to balance overtreatment and infection-related morbidity.

Diagnosing EOS remains challenging due to non-specific clinical signs (e.g., respiratory distress and feeding intolerance) and delayed/inconclusive blood cultures. Up to 30% of EOS cases exhibit no initial symptoms (7), while traditional biomarkers, such as leukocyte counts lack sensitivity (area under curve: AUC < 0.65) (8). The EOS Risk Calculator is a newborn EOS risk prediction model developed by foreign scholars. It can accurately predict the risk of EOS in newborns and provide recommendations for the use of appropriate antimicrobial drugs based on the risk level. This can significantly reduce the rate of blood culture testing and decrease the clinical use of antimicrobial drugs (9). Although risk calculators, such as the Kaiser Permanente EOS calculator, can reduce unnecessary antibiotics by 40%–60% in Western cohorts (10), their applicability in China remains underexplored, particularly in settings with a high prevalence of maternal fever and heterogeneous etiologies, such as epidural-related and infectious cases. At our high-volume maternity center (>10,000 annual deliveries), 8%–12% of parturients develop intrapartum fever, necessitating evaluating NICU evaluation, equivalent to 800–1,200 neonates annually.

It is important to acquire additional safety data, and there is a lack of information regarding the EOS risk calculator application in the management of risk population in developing nations. Specifically, prior to its implementation in China, we aimed to determine whether this procedure could effectively diminish the administration of antibiotics within our unit by analyzing existing data. In addition, we evaluated the predictive accuracy of a combination of the EOS calculator and traditional blood biomarkers in cases of culture-positive sepsis, culture-negative sepsis, and uninfected neonates. To address overtreatment (40% of patients receive empirical antibiotics) and optimize the use of resources, we piloted a novel protocol integrating the EOS calculator with dynamic inflammatory markers [C-reactive protein (CRP) and Interleukin 6 (IL-6)], pathogen cultivation and placental pathology. We hypothesized that this combined approach would enhance diagnostic accuracy while maintaining neonatal safety, thus providing insights for antibiotic stewardship in resource-constrained settings.

## 2 Materials and methods

### 2.1 Study design and data collection

This retrospective cohort study included 265 term neonates (gestational age: 37–41⁶/₇ weeks) born between the 1st of January 2024 and the 30th of June 2024 at the International Peace Maternity and Child Health Hospital in Shanghai China. Eligible neonates were admitted to the NICU for evaluation or treatment due to maternal intrapartum fever (temperature >37.5 °C). Clinical data were extracted from electronic medical records, encompassing maternal and neonatal demographics (sex, delivery mode, birth weight, gestational age), laboratory parameters [serial white blood cell (WBC) and CRP levels at 12–48 h], intrapartum factors [maternal fever peak, epidural anesthesia use, premature rupture of membranes (PROM) duration, amniotic fluid characteristics], placental pathology, and microbiological results [umbilical cord blood cultures, maternal group B Streptococcus (GBS) colonization]. For neonates, umbilical cord blood was collected at birth for microbial culture and procalcitonin (PCT). Peripheral venous blood was drawn 4–6 h after birth for complete blood count (CBC) and C-reactive protein (CRP). For mothers, peripheral venous blood was collected within 2 h of fever onset for CBC, CRP, and IL-6. Neonates were excluded if they were transferred to other hospitals, had congenital anomalies, were from multiple pregnancies, or had mothers with confirmed non-obstetric fever (e.g., respiratory infections). During delivery or after membrane rupture, cervical or endometrial secretions were collected using a dedicated sterile swab by the attending physician and immediately sent for aerobic and anaerobic microbial culture. This study was approved by the Institutional Review Board of the International Peace Maternity and Child Health Hospital, Shanghai, China (Approval number: 202416); the requirement for informed consent was waived.

### 2.2 Diagnostic criteria

EOS was diagnosed according to the 2024 Chinese Expert Consensus on Neonatal Sepsis (7), as follows. A suspected diagnosis was defined as clinical abnormalities (e.g., respiratory distress, poor perfusion) within 72 h postpartum and/or the presence of major EOS risk factors (e.g., maternal chorioamnionitis). A clinical diagnosis was defined as clinical abnormalities plus ≥ 2 positive non-specific laboratory criteria [abnormal WBC, CRP, IL-6 or procalcitonin (PCT)]. A confirmed diagnosis was defined as clinical abnormalities with positive blood or cerebrospinal fluid cultures or the detection of pathogenic DNA. EOS was defined as sepsis occurring ≤3 days postpartum or ≤7 days for GBS infections.

Placental pathology was categorized using Naeye's staging system based on neutrophil infiltration (11) to define histologic chorioamnionitis (HCA) (as reported by our pathology department during the study period): Stage I {scattered neutrophils [< 10/high-power field (HPF)] in subchorionic fibrin}; Stage II [moderate infiltration (11–30/HPF) extending to chorionic tissue or fetal vessels]; or Stage III (extensive inflammation (>30/HPF) involving the amnion.

### 2.3 Group stratification and study outcomes

**Figure 1:**
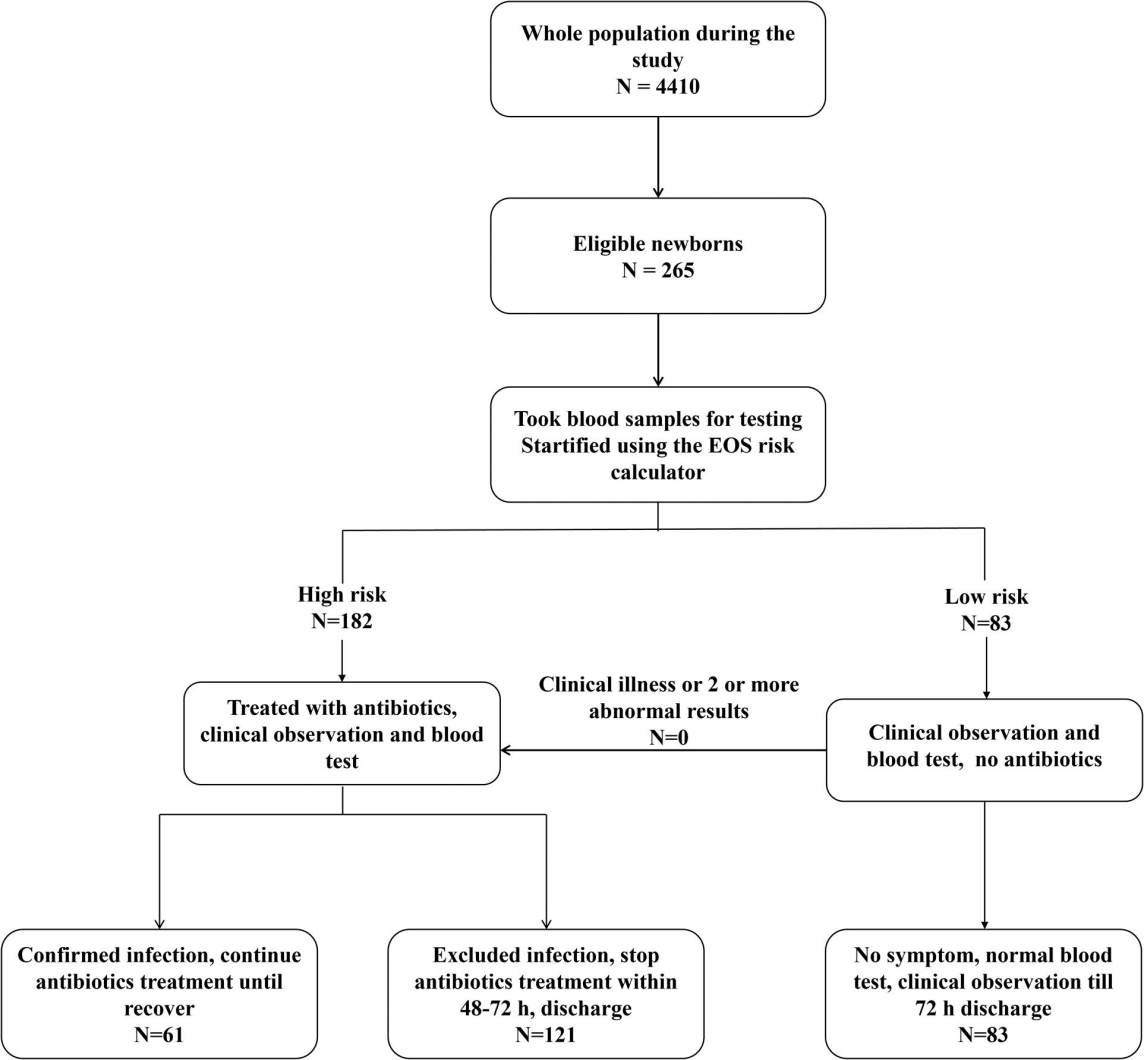
Flowchart of newborns in the study.

Neonates were stratified using the EOS Risk Calculator (10) incorporating maternal PROM duration, GBS status, amniotic fluid characteristics, and WBC/platelet counts. Low-risk neonates underwent clinical observation only, while high-risk neonates received empiric antibiotics. Primary outcomes included the rates of culture-proven or clinically diagnosed EOS, antibiotic utilization patterns, and biomarker performance (CRP, IL-6) for the prediction of infection (Figure 1).

Investigators reviewed the neonates' medical charts and collected data on maternal risk factors. These risk factors [including the highest maternal antepartum temperature, gestational age, premature rupture of membranes (PROM), maternal colonisation with group B streptococci (GBS) and type and timing of intra-partum antibiotic therapy] were entered into the EOS risk calculator to determine a baseline risk of EOS at birth. Next, investigators classified each neonate as “well appearing”, “equivocal”, or with “clinical illness” using the clinical signs body temperature, heart rate, respiratory rate etc., Supplementary Table S1) described on the Kaiser Permanente website (https://neonatalsepsiscalculator.kaiserpermanente.org, 2017 version). Each neonate's EOS risk was determined using the EOS risk calculator, with the “incidence of early-onset sepsis” variable set at “0.6/1000 live births.” If the clinical recommendations given by the EOS calculator included empirical antibiotics, the neonate was classified as high risk; if no antibiotics were recommended, the neonate was classified as low risk. See the flowchart for details (Figure 1).

### 2.4 Statistical analysis

Data were analyzed using IBM SPSS Statistics 29.0.1.0 (171) (Cabit Information Technology Co., Ltd, Shanghai, China). The normality of the data was assessed with the Kolmogorov–Smirnov test. Normally distributed continuous variables are expressed as mean ± standard deviation (SD) and compared with the student's *t*-test or analysis of variance (ANOVA). Non-parametric data {median [inter-quartile range (IQR)]} were analyzed by Kolmogorov–Smirnov or Kruskal–Wallis tests. Categorical variables were compared using *χ*^2^ or Fisher's exact tests. Multivariate logistic regression was then used to identify risk factors for EOS, and receiver operator characteristic (ROC) curves were used to evaluate the diagnostic performance of biomarkers. A two-tailed *P* < 0.05 denoted statistical significance.

## 3 Results

### 3.1 Baseline characteristics of the study population

Of the 265 neonates born to mothers with intrapartum fever, 61 (23.0%) were classified into the infected group based on confirmed or clinically diagnosed early-onset sepsis (EOS), while 204 (77.0%) were classified into the uninfected group (Table 1). Of the 61 neonates in the infected group, only 3 (4.9%) had culture-proven sepsis (2 Group B Streptococcus, 1 E. faecalis), while the remaining 58 were culture-negative but met criteria for clinical EOS, reflecting the typically low blood culture positivity rate in neonatal EOS. No significant differences were observed between the two groups in terms of gestational age (median: 39.5 vs. 39.6 weeks, *P* = 0.26), birthweight (3,375 g vs*.* 3,335 g, *P* = 0.23), sex distribution (50% vs. 55% male, *P* = 0.32), cesarean section rates (29% vs. 18%, *P* = 0.07), or maternal fever peak (median 38.0 °C vs. 38.0 °C, *P* = 0.24). Similarly, there were no significant differences between the two groups in terms of PROM (≥18 h, 16% vs. 16%, *P* = 0.97) and maternal GBS colonization (2% vs*.* 3%, *P* = 0.91).

**Table 1:** Baseline characteristics of neonates and mothers.

Clinical characteristics	Uninfected (*n* = 204)	Infected (*n* = 61)	*P*
Neonates			
Gestational age, week, median (IQR)	39.5 (38.6, 40.3)	39.6 (38.8, 40.3)	0.26
Birthweight, g, median (IQR)	3,375 (3,100, 3,593)	3,335 (3,045, 3,550)	0.23
Male gender, *n* (%)	102 (50%)	34 (0.55)	0.32
Caesarean section, *n* (%)	60 (29%)	11 (18%)	0.07
epidural anesthesia, *n* (%)	166 (81%)	54 (88%)	0.23
Meconium-stained amniotic fluid, *n* (%)	13 (6%)	20 (32%)	0.001
Duration of ROM more than18 h, *n* (%)	33 (16%)	10 (16%)	0.968
Neonatal clinical illness (*Respiratory failure needs oxygen therapy; Jaundice needs phototherapy; Feeding intolerance)*	2 (1%)	40 (65%)	0.001
Mothers			
Maternal fever Tmax median (IQR)	38 (37.9, 38.2)	38 (37.8, 38.1)	0.24
maternal GBS colonization, *n* (%)	6 (2%)	2 (3%)	0.907
Suspected chorioamnionitis, *n* (%)	100 (49%)	32 (52%)	0.31
Duration of antibiotics, median (IQR)	3 (0, 3)	5 (5, 7)	0.001

IQR, Interquartile range; ROM, Rupture of membranes; GBS, Group B streptococcus.

Significant differences were detected between the two groups in terms of the characteristics of the amniotic fluid and the clinical condition of the neonates. Meconium-stained or odorous amniotic fluid was five-fold more common in infected newborns than uninfected newborns (32% vs. 6%; *P* < 0.001). Furthermore, abnormal clinical signs in neonates, such as respiratory distress and lethargy, were present in 65% of infected newborns; this compared to only 1% of uninfected neonates (*P* < 0.001).

### 3.2 Maternal-Neonatal inflammatory dynamics and placental pathology

Analysis of neonatal laboratory markers revealed that infected neonates had significantly higher levels of inflammatory markers when compared to their uninfected peers. Specifically, the median percentage of neutrophils was 72.6% (IQR: 67.8–75.4) in the infected group; this compared to 67.9% (63.1–72.6) in the uninfected group (*P* < 0.001 (Table 2). Furthermore, the median CRP level as 11.5 mg/L (7.5–22.5) in infected neonates compared to 3.5 mg/L (2–6) in uninfected neonates (*P* < 0.001). Median IL-6 levels were significantly higher in infected neonates than uninfected neonates (98.3 pg/mL (23.7–535) vs*.* 18.7 pg/mL (11.1–53.6) (*P* < 0.001). Furthermore, median PCT levels were 0.20 μg/L (0.12–0.66) in the infected group compared to 0.13 μg/L (0.10–0.16) in the uninfected group (*P* = 0.003). Moreover, blood culture positivity was infrequent, occurring in only 5% of infected infants compared to none in the uninfected group (*P* = 0.01). In contrast, serial WBC counts, measured at multiple time points, did not differ significantly between groups, with median values of 22.1 vs*.* 22.3 × 10⁹/L (*P* = 0.97).

**Table 2:** Comparison of maternal-neonatal laboratory markers and placental findings.

Serum markers and placental pathology	Uninfected (*n* = 204)	Infected (*n* = 61)	*P* value
Baby			
WBC (10^9^/L) Median (IQR)	22.3 (18.5, 26.3)	22.1 (18.4, 26.9)	0.973
Segment (%)	67.9 (63.1, 72.6)	72.6 (67.8, 75.4)	<0.001
CRP (mg/L)	3.5 (2, 6)	11.5 (7.5, 22.5)	<.0001
PCT (μg/L)	0.13 (0.10, 0.16)	0.20 (0.12, 0.66)	0.003
IL-6	18.7 (11.1, 53.6)	98.3 (23.7, 535)	<0.001
Blood culture positive, *n* (%)	0 (0)	3 (5%)	0.01
Maternal			
WBC (10^9^/L)	14.6 (12.5, 16.9)	15.7 (12.2, 18.4)	0.22
Segment (%)	85.5 (82.9, 88)	86.6 (84.4, 89)	0.13
CRP (mg/L)	10 (6, 16)	13 (7, 28)	0.007
PCT (μg/L)	0.06 (0.04, 0.10)	0.06 (0.03, 0.08)	0.07
IL-6	248.5 (89.1, 1,448.2)	298.5 (93.8, 3,188)	<0.001
Maternal intrauterine cavity cultures positive, *n* (%)	50 (24%)	25 (41%)	0.012
Histological chorioamnionitis	135	43	0.52

WBC, White blood cell; IQR, Interquartile range; CRP, C-reactive protein; PCT, Procalcitonin; IL-6, Interleukin 6.

Analysis of the maternal inflammatory profile showed that the levels (median) of maternal CRP were significantly elevated in the infected group, with levels of 13 mg/L compared to 10 mg/L in the uninfected group (*P* = 0.007). Additionally, median IL-6 levels were significantly higher in the infected group (298.5 pg/mL) compared to the uninfected group (248.5 pg/mL) (*P* < 0.001). However, there was no significant difference in WBC counts; WBC count was 15.7 × 10⁹/L in the infected group compared to 14.6 × 10⁹/L in the uninfected group (*P* = 0.22). Similarly, the percentage of neutrophils did not differ significantly between the two groups (86.6% vs*.* 85.5%; *P* = 0.13).

Placental microbiology and histology studies revealed that positive intrauterine cultures were observed more frequently in the infected cohort, with a rate of 41% compared to 24% in the non-infected cohort (*P* = 0.012). Notably, there was an 83% concordance rate between maternal intrauterine and neonatal blood bacterial isolates (Table 2). In contrast, the incidence of histological chorioamnionitis was similar in both cohorts, with rates of 66% and 70%, respectively (*P* = 0.52), regardless of the fetal inflammatory response (FIR) grade. To further characterize the infected cohort, detailed clinical outcomes and complications are summarized in Table 3. Among the 61 neonates with EOS, the median duration of antibiotic therapy was 7 days, with 3 culture-positive cases. No cases of IVH, NEC, or death were observed.

**Table 3:** Outcomes and characteristics of infected babies (*n* = 61).

Outcomes and characteristics	Results
Length of stay (days, median IQR)	7 (5,9)
Duration of antibiotics (days, median IQR)	5 (5,7)
Positive results of blood culture (*n*)	3 (5%)
Respiratory failure needs oxygen therapy (*n*, %)	21 (34%)
Jaundice needs phototherapy (*n*, %)	18 (29%)
NEC (*n*)	0
Intracranial infection (*n*)	0
Sepsis shock (*n*)	0
Death (*n*)	0

IQR, interquartile range; NEC, necrotizing; 3 positive results of blood culture (2 group B streptococcus,1 E. faecalis).

### 3.3 Diagnostic performance of inflammatory biomarkers

**Figure 2:**
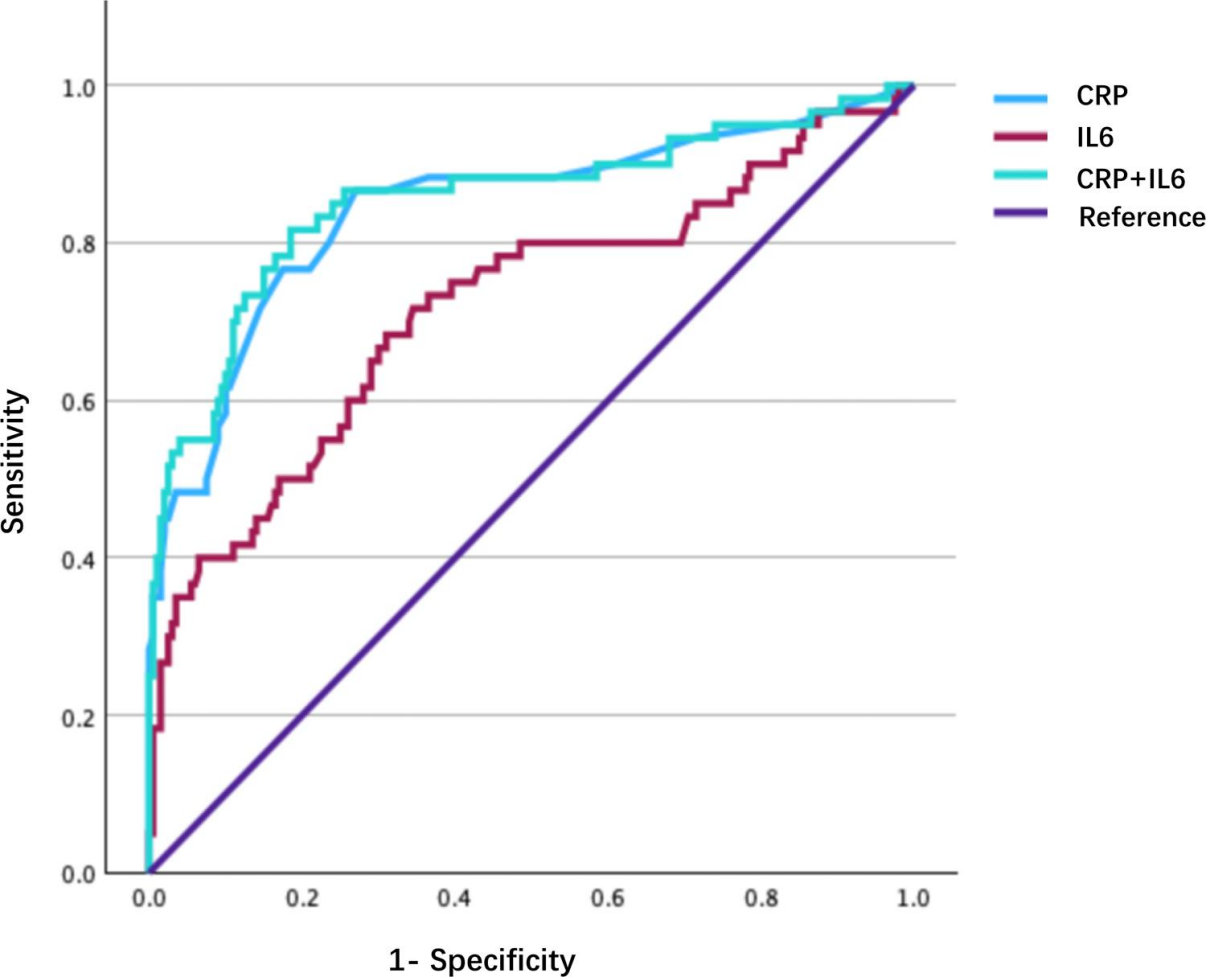
ROC curves for CRP, IL-6 and their combination. ROC, Receiver Operating Characteristic; CRP, C-reactive protein; IL-6, Interleukin 6.

As illustrated in Figure 2, ROC curve analysis revealed that CRP and IL-6 emerged as the foremost predictors of neonatal infection. Notably, CRP exhibited the highest discriminatory ability, with an AUC of 0.84 [95% confidence interval (CI): 0.78–0.91] with a sensitivity of 82% and a specificity of 89% at the optimal cutoff value of 7.8 mg/L. Additionally, IL-6 demonstrated moderate accuracy, attaining an AUC of 0.72 (95% CI: 0.64–0.80) with a sensitivity of 75% and specificity of 68% using a cutoff value of 23.5 pg/mL. Furthermore, the combination of CRP and IL-6 provided a slight enhancement in diagnostic performance. This combination resulted in an AUC of 0.85 (95% CI: 0.79–0.92) and a negative predictive value (NPV) of 93.7%, as detailed in Table 4. Pairwise comparisons of the ROC curves using DeLong's test are presented in Supplementary Table S2. These comparisons showed that CRP was significantly superior to IL-6 alone, and the combination was significantly superior to IL-6 alone, but there was no statistically significant difference between the combination of CRP and IL-6 and CRP alone. In contrast, WBC counts and PCT exhibited limited diagnostic efficacy, with AUC values of 0.51 (*P* = 0.84) and 0.63 (*P* = 0.08), respectively. As shown in Table 5, the predictive values of early-onset sepsis (EOS) calculator demonstrated a PPV of 33.5%, NPV of 100%, sensitivity of 100%, and specificity of 41%.

**Table 4:** ROC curve analysis of inflammatory biomarkers.

Variable	AUC	SD^a^	Approaching Significance^b^	Approaching 95% CI	
				Lower	Upper
CRP	0.844	0.033	0.000	0.779	0.910
IL-6	0.719	0.042	0.000	0.638	0.801
Combination	0.854	0.033	0.000	0.789	0.919

^a^ Under non-parametric assumptions.

^b^ Null hypothesis: the true area = 0.5.

ROC, Receiver Operating Characteristic; AUC, Area Under Curve; SD, Standard deviation; CRP, C-reactive protein; IL-6, Interleukin 6; CI, Confidence interval.

**Table 5:** Predictive values of early-onset sepsis (EOS) calculator.

Predictive value	Results
Correctly classified to infected (*n*)	61
Correctly classified to uninfected (*n*)	83
Sensitivity, %	100%
Specificity, %	41%
PPV, %	33.50%
NPV, %	100%

## 4 Discussion

In this study, we systematically evaluated the integration of the EOS risk calculator with clinical manifestations, inflammatory biomarkers and microbiological evidence to optimize neonatal management in the context of intrapartum maternal fever. Our findings highlight the diagnostic potential of combining risk stratification tools with targeted laboratory evaluations, while highlighting the nuanced relationship between maternal fever, placental pathology and neonatal outcomes.

EOS refers to sepsis occurring within 72 h of birth in newborns, typically caused by amniotic fluid contamination or vertical transmission during vaginal delivery Intrapartum maternal fever is considered a risk factor for EOS (12). Traditionally, the clinical diagnosis of neonatal EOS has been confirmed based on clinical manifestations and laboratory test results, which although can achieve high accuracy, often require a lengthy waiting period, potentially missing the optimal treatment window (13). With advancements in medical technology, improving the efficiency of early diagnosis for EOS has become a key focus of clinical attention and research. The development and application of an EOS risk calculator have provided new opportunities and options for the diagnosis and management of EOS.

The EOS risk calculator demonstrated strong reliability for the identification of low-risk neonates, with only two cases of jaundice observed in the low-risk cohort, due to hemolytic factors. This aligns with large-scale validation studies confirming that the NPV for the calculator exceeded 95% in term neonates (10). Notably, none of the low-risk neonates, classified based on calculator criteria, developed culture-proven sepsis, thus reinforcing the safety of withholding empirical antibiotics in this subgroup. In infected neonates, respiratory distress (65%) and jaundice (28%) were the most common clinical presentations. However, these non-specific symptoms overlap considerably with non-infectious conditions, such as transient tachypnea and hemolytic disease, thus highlighting the limitations of symptom-based diagnosis and the necessity for objective biomarkers. Of the inflammatory markers tested, CRP (AUC = 0.84) outperformed IL-6 (AUC = 0.72) and PCT (AUC = 0.68), consistent with its role as a late-phase reactant in neonatal sepsis. The combination of CRP and IL-6 further enhanced diagnostic accuracy (AUC = 0.85), thus supporting their tandem use to reduce diagnostic uncertainty. As shown in Supplementary Table S2, pairwise comparisons using DeLong's test confirmed that CRP was significantly better than IL-6 (*P* = 0.02) and that the combination was significantly better than IL-6 alone (*P* = 0.01). However, the improvement from adding IL-6 to CRP was modest and did not reach statistical significance (*P* = 0.83). This indicates that, in the current cohort, CRP is the primary biomarker driving diagnostic performance.

Previous analysis of microbial dynamics and placental discordance revealed significant insights into the relationship between maternal infections and neonatal outcomes. Although the prevalence of HCA did not differ between the infected and uninfected groups, neonates in the infection group exhibited significantly higher rates of positive umbilical cord blood cultures (5% compared to 0%, *P* < 0.01) and maternal intrauterine cavity cultures (41% vs. 24%, *P* = 0.012); these results suggested that ascending infection may be the primary pathway for pathogen transmission (14), as evidenced by the strong concordance between intrauterine and neonatal pathogens (*κ* = 0.82), indicating that pathogens likely migrate from contaminated amniotic fluid (15). Positive maternal intrauterine cultures were more frequent in the infected group and may serve as a supportive risk factor in settings where such sampling is performed, though this is not standard of care. Furthermore, a previous study revealed that the decoupling of microbial invasion and placental inflammation; despite differing microbial loads, the absence of disparities in HCA and FIR suggested that histopathological changes may occur after microbial colonization, which could explain why nearly 30% of cases with positive intrauterine cultures did not exhibit placental inflammation. Additionally, a subset of cases with sterile inflammation was noted, as some non-infected neonates presented with maternal fever despite normal biomarkers and cultures, thus indicating potential non-infectious causes such as epidural-related hyperthermia (16), which may require different management strategies.

Moving towards precision antibiotic stewardship, our implementation of a calculator-laboratory marker strategy successfully reduced antibiotic exposure by 31% (85 out of the 265 infants planned to receive treatment were exempted); yet, over half of the treated neonates (121 out of 182) were later determined to be free of infection, thus highlighting the issue of overtreatment. To enhance precision in treatment, we propose several strategies. First, we should establish risk-adapted biomarker thresholds for “indeterminate-risk” neonates via serial CRP measurements at 24 and 48 h to inform decisions relating to the escalation or de-escalation of treatment. Second, we should implement intrauterine culture-guided therapy, in which neonates born to mothers with positive intrauterine cultures should undergo prioritized sepsis evaluations, even if biomarkers are equivocal. Third, we should consider time-limited empirical therapy, such as the administration of short-course antibiotics (e.g., 48 h) while awaiting culture results, to strike a balance between safety and effective antimicrobial stewardship.

Our study has several limitations that should be acknowledged. Firstly, there was an under-representation of cases with high-grade fever, as only four neonates (1.5%) were born to mothers with temperatures exceeding 38.5 °C. This reflects the aggressive intrapartum interventions typically employed in such cases, which limits our ability to analyze the correlation between fever severity and outcomes. However, this does mirror real-world scenarios where low-grade fevers are more common (17). Secondly, we did not account for the timing of antibiotic administration; prophylactic intrapartum antibiotics given to GBS-positive mothers may have suppressed the yields of umbilical blood cultures, potentially leading to the underestimation of true infection rates (18). Furthermore, our placental sampling may have been incomplete, as focal chorioamnionitis lesions could have been missed despite adherence to standardized placental examination guidelines (19). Nonetheless, the predominance of mild to moderate maternal fever (<38.5 °C) in our cohort aligns with clinical reality, thereby enhancing the external validity of our findings. However, we must acknowledge that there are many factors influencing fever during labor, such as labor duration, the number of vaginal examinations, and the use of epidural analgesia. In this study, no difference in the rate of epidural analgesia use among mothers was found between the infected and non-infected groups. However, data on labor duration and other relevant factors were not collected or analyzed, which should be addressed in future research. Importantly, our study advances beyond the traditional focus on maternal fever alone by incorporating placental microbiology and cytokine profiling, specifically the measurement of IL-6 and CRP levels.

Looking ahead, three key priorities emerge for future research: first, the integration of rapid diagnostics, such as polymerase chain reaction (PCR)-based pathogen detection, could complement traditional cultures and expedite neonatal management (20). Second, dynamic cytokine mapping is warranted, as the early increase of IL-6 (within 6–8 h) (21) compared to the delayed peak of CRP (24–48 h) (22) suggests that sequential monitoring protocols may be beneficial for high-risk cases. Finally, investigating behavioral fever phenotyping to distinguish between infectious and non-infectious maternal fever through advanced biomarkers, such as presepsin and the CD64 index (23, 24), is a promising avenue for further investigation.

## 5 Conclusion

Combining clinical manifestations, inflammatory biomarkers, and microbiological evidence, the EOS risk calculator has good diagnostic value for suspected early-onset sepsis, significantly reducing antibiotic usage rates while improving accuracy and safety. It provides a basis for the early identification and treatment of neonatal EOS, promoting the rational use of medical resources.

## Data Availability

The original contributions presented in the study are included in the article/Supplementary Material, further inquiries can be directed to the corresponding author.
