# Supplementary material for: Optimizing neonatal management in the prescence of intrapartum maternal fever via the integration of an early-onset sepsis risk calculator and dynamic inflammatory markers
Source: Front Pediatr. 2026 Feb 11;14:1670738. doi: 10.3389/fped.2026.1670738 (PMC12932430; doi:10.3389/fped.2026.1670738)
Supplement: Supplementary file 1 [file Table1.docx]

**Table S1. Classification of Infant's Clinical Presentation**

https://neonatalsepsiscalculator.kaiserpermanente.org/Classification.aspx

| **Clinical Exam** | **Description** |
| --- | --- |
| **Clinical Illness** | 1. Persistent need for NCPAP / HFNC / mechanical ventilation (outside of the delivery room) 2. Hemodynamic instability requiring vasoactive drugs 3. Neonatal encephalopathy /Perinatal depression    - Seizure    - Apgar Score @ 5 minutes < 5 4. Need for supplemental O_2_ > 2 hours to maintain oxygen saturations > 90% (outside of the delivery room) |
| **Equivocal** | 1. Persistent physiologic abnormality > 4 hrs    - Tachycardia (HR > 160)    - Tachypnea (RR > 60)    - Temperature instability (> 100.4˚F or < 97.5˚F)    - Respiratory distress (grunting, flaring, or retracting) not requiring supplemental O_2_ 2. Two or more physiologic abnormalities lasting for > 2 hrs    - Tachycardia (HR > 160)    - Tachypnea (RR > 60)    - Temperature instability (> 100.4˚F or < 97.5˚F)    - Respiratory distress (grunting, flaring, or retracting) not requiring supplemental O_2_   Note: abnormality can be intermittent |
| **Well Appearing** | No persistent physiologic abnormalities |

**Table S2.** Pairwise comparisons of ROC curves for inflammatory biomarkers using DeLong's test.

|  | z | P (2- tail) |
| --- | --- | --- |
| IL6 vs CRP | 2.35 | 0.02 |
| CRP vs CRP+IL6 | 0.21 | 0.83 |
| IL6 vs CRP+IL6 | 2.54 | 0.01 |
